# *Rickettsia raoultii*–like Bacteria in *Dermacentor* spp. Ticks, Tibet, China

**DOI:** 10.3201/eid1809.120644

**Published:** 2012-09

**Authors:** Yuefeng Wang, Zhijie Liu, Jifei Yang, Ze Chen, Jianzhi Liu, Youquan Li, Jianxun Luo, Hong Yin

**Affiliations:** SKLVEB, Lanzhou Veterinary Research Institute, Lanzhou, Gansu, China (Y. Wang, Z. Liu, J. Yang, Z. Chen, Y. Li, J. Luo, H. Yin);; and Tibet Livestock Research Institute, Lhasa, Tibet, China (J. Liu)

**Keywords:** rickettsia, Rickettsia raoultii, R. raoultii–like bacteria, Candidatus Rickettsia tibetani, bacteria, spotted fever group, ticks, Dermacentor everestianus, Dermacentor niveus, Tibet, China

**To the Editor:** *Rickettsia raoultii* is an obligate intracellular gram-negative bacterium belonging to the spotted fever group (SFG) of the genus *Rickettsia*. Genotypes RpA4, DnS14, and DnS28, originally isolated from ticks from Russia in 1999 (*1*), were designated as *Rickettsia raoultii* sp. nov. on the basis of phylogenetic analysis (*2*). *R. raoultii* has been found mainly in *Dermacentor* spp. ticks in several countries in Europe (*3*). It was detected in a *Dermacentor marginatus* tick from the scalp of a patient with tick-borne lymphadenitis in France (*2*), which suggests that it might be a zoonotic pathogen. We determined the prevalence of *R. raoultii*–like bacteria in *Dermacentor* spp. in highland regions in Tibet.

Ticks from sheep (*Ovis aries*) near Namuco Lake (a popular tourist destination 4,718 m above sea level) were collected and identified morphologically as *D. everestianus* and *D. niveus* ticks (*4*). Genomic DNA was extracted from individual specimens by using the QIAamp DNA Mini Kit (QIAGEN, Hilden, Germany). All DNA samples were amplified by using PCRs specific for the citrate synthase (*gltA*, 770 bp) gene (*5*) and the outer membrane protein A (*ompA*, 629 bp) gene (*6*). Some samples were amplified by using a PCR specific for the *ompB* (2,479 bp) gene (*7*).

Randomly selected amplicons for *gltA* (n = 27), *ompA* (n = 31), and *ompB* (n = 7) were cloned into the pGEM-T Easy vector (Promega, Shanghai, China) and subjected to bidirectional sequencing (Sangon Biotech, Shanghai, China). Sequences obtained were deposited in GenBank under accession nos. JQ792101–JQ792105, JQ792107, and JQ792108–JQ792166. Phylogenetic analysis was conducted for sequences we identified and sequences of recognized SFG rickettsial species available in Genbank by using the MegAlign program (DNASTAR, Inc., Madison, WI, USA) and MEGA 4.0 (*8*).

Of 874 tick specimens, 86 were *D. everestianus* ticks (13 male and 73 female), and 788 were *D. niveus* ticks (133 male and 655 female). Samples positive for *gltA* and *ompA* were considered SFG rickettsial species. Using this criterion, we found that 739 tick specimens (84.6%) were positive for *Rickettsia* spp. Of 86 *D. everestianus* ticks, 85 (98.8%) were positive for *Rickettsia* spp. and of 788 *D. niveus* ticks, 654 (83.0%) were positive. Infection rates for male and female *D. niveus* ticks were 87.9% and 82.1%, respectively. We found an overall prevalence of 84.6% for *R. raoultii*–like bacteria in *Dermacentor* spp. in the highland regions in Tibet.

Nucleotide sequence identities ranged from 99.2% to 100% (except for isolate WYG55, which had an identity of 98.6%) for the *ompA* gene and from 99.2% to 99.9% (except for isolate XG86, which had an identity of 98.5%) for the *ompB* gene. These results indicated that homology levels of most isolates were within species thresholds (*ompA* ≥98.8% and *ompB* ≥99.2%) (*9*). Isolate WYG55 showed the lowest identity (98.2%) among *gltA* gene sequences and the lowest identity (98.6%) among *ompA* gene sequences. Isolate XG86 showed lowest identity (98.5%) among *ompB* gene sequences. These results suggest that other *Rickettsia* spp. were among the investigated samples.

A BLASTn search (www.ncbi.nlm.nih.gov) for the obtained sequences was conducted. The best matches (highest identities) detected were with sequences of *R. raoultii*. However, comparison of our sequences with corresponding sequences of *R. raoultii* in GenBank showed identity ranging from 98.0% to 99.0% for *ompA* and from 98.1% to 99.0% for *ompB*, which did not meet the threshold (*9*) for *R. raoultii*. We compared the new sequences with corresponding reference sequences of universally recognized SFG group *Rickettsia* spp. in Genbank and constructed 2 phylogenetic trees (Figure). The new sequences were placed into separate branches, which were closely related to *R. raoultii* branches.

**Figure:**
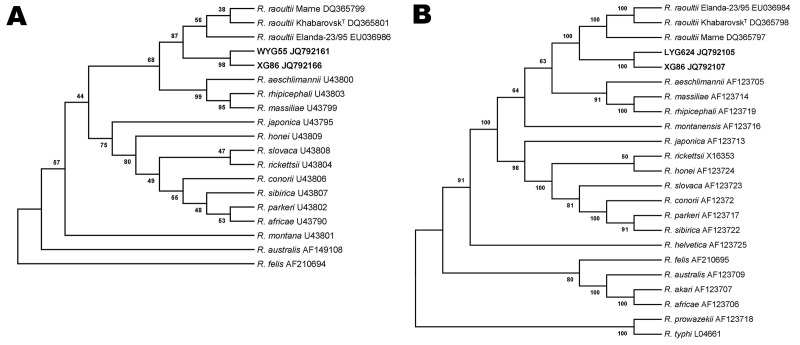
Unrooted phylogenetic trees inferred from comparison of A) outer membrane protein A (*ompA*) and B) *ompB* gene sequences of rickettsial species by using the neighbor-joining method. Sequences in **boldface** were obtained during this study. Numbers at nodes are the proportion of 100 bootstrap resamplings that support the topology shown.

Prevalence of *R. slovaca* and *R. raoultii* was 6.5% and 4.5% in *D. silvarum* ticks in Xinjiang Uygur Autonomous Region of China (*10*). In contrast, we found that the overall prevalence of *R. raoultii*–like bacteria might be ≤84.6% in *D. everestianus* and *D. niveus* ticks in Dangxiong County in Tibet.

Our findings suggest that *D. everestianus* and *D. niveus* ticks are potential vectors of *R. raoultii*–like bacteria and indicate that spread of *R. raoultii*-like bacteria encompasses a large area in China. In the study sites, yak and Tibetan sheep are the major domestic animals, and rodents are the major wild animals. Rodents are also the major hosts of *Dermacentor* spp. ticks, which can transmit *R. raoultii* transstadially and transovarially (*2*). Animals bitten by infected ticks can acquire the pathogen and serve as natural reservoirs.

On the basis of phylogenetic analysis, we found that the *Rickettsia* spp. in ticks investigated represents a novel species, which can be designated *Candidatus* Rickettsia tibetani. However, additional phylogenetic studies are needed to obtain more information on the molecular biology of these bacteria.

## References

[1] Rydkina E, Roux V, Rudakov N, Gafarova M, Tarasevich I, Raoult D. New *Rickettsiae* in ticks collected in territories of the former Soviet Union. Emerg Infect Dis. 1999;5:811–4. 10.3201/eid0506.990612 10603217 PMC2640811

[2] Mediannikov O, Matsumoto K, Samoylenko I, Drancourt M, Roux V, Rydkina E, *Rickettsia raoultii* sp. nov., a spotted fever group *rickettsia* associated with *Dermacentor* ticks in Europe and Russia. Int J Syst Evol Microbiol. 2008;58:1635–9. 10.1099/ijs.0.64952-0 18599708

[3] Spitalská E, Stefanidesova K, Kocianova E, Boldis V. *Rickettsia slovaca* and *Rickettsia raoultii* in *Dermacentor marginatus* and *Dermacentor reticulatus* ticks from Slovak Republic. Exp Appl Acarol. 2012;57:189–7. 10.1007/s10493-012-9539-8 22392435

[4] Teng KF, Jiang ZJ. Economic insect fauna of China, Acari, Ixodidae. Beijing: Science Press; 1991.

[5] Roux V, Rydkina E, Eremeeva M, Raoult D. Citrate synthase gene comparison, a new tool for phylogenetic analysis, and its application for the *rickettsiae.* Int J Syst Bacteriol. 1997;47:252–61. 10.1099/00207713-47-2-252 9103608

[6] Roux V, Fournier PE, Raoult D. Differentiation of spotted fever group *rickettsiae* by sequencing and analysis of restriction fragment length polymorphism of PCR-amplified DNA of the gene encoding the protein rOmpA. J Clin Microbiol. 1996;34:2058–65. 8862558 10.1128/jcm.34.9.2058-2065.1996 PMC229190

[7] Blair PJ, Jiang J, Schoeler GB, Moron C, Anaya E, Cespedes M, Characterization of spotted fever group *rickettsiae* in flea and tick specimens from northern Peru. J Clin Microbiol. 2004;42:4961–7. 10.1128/JCM.42.11.4961-4967.2004 15528680 PMC525230

[8] Tamura K, Dudley J, Nei M, Kumar S. MEGA4: Molecular Evolutionary Genetics Analysis (MEGA) software version 4.0. Mol Biol Evol. 2007;24:1596–9. 10.1093/molbev/msm092 17488738

[9] Raoult D, Fournier PE, Eremeeva M, Graves S, Kelly PJ, Oteo JA, Naming of Rickettsiae and rickettsial diseases. Ann N Y Acad Sci. 2005;1063:1–12. 10.1196/annals.1355.002 16481485

[10] Tian ZC, Liu GY, Shen H, Xie JR, Luo J, Tian MY. First report on the occurrence of *Rickettsia slovaca* and *Rickettsia raoultii* in *Dermacentor silvarum* in China. Parasit Vectors. 2012;5:9. 10.1186/1756-3305-5-19 22257726 PMC3292836

